# Supplementary material for: Are carbon emissions trading and green financial instruments synergistic? -Comprehensive quantitative research based on content analysis
Source: PLoS One. 2024 Mar 7;19(3):e0298601. doi: 10.1371/journal.pone.0298601 (PMC10919601; doi:10.1371/journal.pone.0298601)
Supplement: S1 Data — (ZIP) [file pone.0298601.s001.zip › Data/地级市Z_F工作报告.zip!/║╙─╧/σo╤⌠╩╨/└┤╫╘╣1⁄2╓┌║┼í╛┬φ┐╦╩2╛▌═°í┐_2005.docx]

各位代表：

现在，我代表市人民政府，向大会作政府工作报告，请予审议，并请各位政协委员提出意见。

一、2004年工作回顾

2004年，是我市经济社会发展的重要一年。一年来，市政府在省委、省政府和市委的领导下，认真贯彻落实党的十六大和十六届三中、四中全会精神，牢固树立和落实科学发展观，坚持“五个统筹”，深入实施以工兴市、科教兴濮、对外开放、可持续发展和品牌城市战略，调整经济结构，转变经济增长方式，以新型工业化为核心，大力发展非公有制经济和县域经济，促进了濮阳经济社会快速发展。特别是针对上半年我市经济发展出现的新情况，采取一系列超常规措施，加压驱动，主攻发展中的主要矛盾，使全市的经济发展蒸蒸日上，百舸争流，圆满完成了市五届人大一次会议确定的各项任务，经济发展实现了超过全省平均水平的奋斗目标，被省政府授予2004年度目标管理优秀单位。

———经济发展全面提速，人均水平上了一个新台阶。初步核算，2004年全市生产总值达到319.8亿元，比上年增长15.8%，比全省平均增幅高2.1个百分点，是1995年以来的最高增幅。人均生产总值突破1000美元大关，达到1100美元。这标志着濮阳经济在新一轮发展中步入了快速发展的新时期。

——工业经济快速增长，质量效益显著提高。全市工业完成增加值153.9亿元，比上年增长20.3%，其中地方工业完成增加值82.3亿元，比上年增长28.3%。全市规模以上工业完成增加值120.4亿元，增长20.5%，其中地方规模以上工业完成增加值48.8亿元，增长35%。规模以上工业经济效益综合指数达到168.9%，比上年提高25.4个百分点；实现销售收入371.7亿元，比上年增长28.4%；实现利税43.5亿元，增长82%；盈亏相抵后实现利润29.2亿元，增长170.2%。年初确定的90个重点工业项目，完成投资66.9亿元，占年计划的124%。有67个项目竣工投产，创造就业岗位1.2万个，年新增产值33亿元、利税5亿元。

———粮食生产获得丰收，农村经济稳定发展。全市粮食总产达到196.7万吨，比上年增长22.7%，是历史上第三个丰收年。农业结构调整成效显著，完成155万亩优质专用小麦、38万亩优质水稻、90万亩优质玉米、30万亩出口型优质花生、5000亩鲜切花基地的开发和配套建设。经济作物面积达到236.8万亩，初步建成了尖椒、冬枣、葡萄、无公害蔬菜等一批名优特新农产品基地。畜牧养殖业迅速发展，全市肉类总产达到21.1万吨、禽蛋总产达到25.9万吨、奶类总产达到1.6万吨，分别比上年增长5.2%、5.8%和35.2%。农业产业化稳步推进，全市各类产业化龙头企业达到355家，其中年销售额超亿元的8家。农田水利基本建设成绩显著，夺魁全省“红旗渠精神杯”市级杯五连冠。林业生态建设取得新成绩。扶贫开发工作力度进一步加大，年内实现了4.5万人脱贫。

———重点项目建设实现突破，经济发展后劲增强。全社会固定资产投资完成126.5亿元，比上年增长27%，首次突破百亿元大关。其中市地方固定资产投资累计完成76.5亿元，增长48.4%。全市142个重点项目，完成投资92.9亿元，占年计划投资的116.3%，有85个项目竣工投产。尤其是关系濮阳发展大局的重点项目建设实现了新突破。濮鹤高速公路建成通车，汤台铁路改造竣工扫尾，林纸项目制浆工程主装置和设备安装基本完成，林纸一体化项目得到国家发改委批复立项。世锦园二期工程建成投用，垃圾发电项目基本建成。濮范高速公路可研报告经省发改委立项批复。濮信高速公路濮阳段、孙口黄河大桥、2×20万千瓦热电机组工程、中原大化3万吨三聚氰胺工程、范县陆集村台建设进展顺利。在完善了村村通柏油路基础上，实现了村村通班车，全市建成21个乡镇汽车站。濮阳职业技术学院基本建成，在校生超过8000人。

———改革开放力度加大，招商引资成效显著。全市210家国有企业改制面达到91.4%，其中去年完成改制25户，盘活资产3.25亿元，安置职工6035人。市三化、甲醇厂等一批企业，通过股份制改造、兼并、租赁经营等形式，增强了活力，取得了良好的效益。市一机厂、特种电缆厂依法完成了破产。同时利用政策，积极推进建市以来国有企业和行政事业单位的不良债务重组工作，并取得实质性进展。认真贯彻《行政许可法》，对行政审批项目进行了清理，审批项目由2003年的353项压减到206项。完成了新一轮市直和县（区）机构改革任务。事业单位人事制度改革取得成效，全市事业单位初步建立了全员聘用的用人制度。事业单位与职工全部签定了聘用合同。粮食流通体制改革和农村信用社体制改革取得阶段性成果。按照中央和省要求，基本完成了中央企业分离办社会工作任务，有力地支持了大企业的发展。加大招商引资力度，精心组织了赴“长三角”、“珠三角”招商引资活动，签定项目52个，其中合同项目4个，协议项目24个，意向项目24个，总投资31.2亿元。赴东南亚参加了世界舜裔宗亲联谊第十八届大会，并开展招商活动，国家批准了我市与吉尔吉斯斯坦楚河地区缔结友好城市关系，扩大了对外交流与合作。内外贸易迅速发展，全市社会消费品零售总额达到84.6亿元，比上年增长15.5%。外贸进出口总额达到1.23亿美元，增长63.8%，其中外贸出口首次突破1亿美元大关，增长47.2%。全年实际到位市外资金21.3亿元人民币，其中实际利用外资2925万美元，比上年增长25%。累计新签对外承包工程和外派劳务合同额2.25亿美元，比上年增长71.3%；外派人数达2254人，增长37.4%。外经工作在全省继续保持领先地位。

———非公有制经济迅速发展，成为新的经济增长点。全市非公有制经济实体达到8.6万家，从业人员48.3万人。完成增加值135.3亿元，比上年增长18.9%；实现营业收入546亿元，增长18.5%；交纳税金7.4亿元，增长19.8%。全市新上投资千万元以上的非公有制工业项目81个。非公有制经济占全市经济总量的43%。

———财政收入增加，金融稳定运行。全市财政收入完成14.5亿元，比上年增长26.9%。其中一般预算收入完成12.6亿元，增长24.7%。全市财政支出完成27.9亿元,比上年增长18.1%。其中一般预算支出完成25.5亿元，增长13.2%。通过召开银企联谊会、重点项目建设协调会等形式，金融机构加大了对地方经济的支持力度。全市金融机构各项存款余额达到277亿元，比年初净增25.9亿元。各项贷款余额达到142.7亿元，净增6.9亿元，剔除债转股、不良贷款剥离等因素的影响，实际新增14.3亿元。

———城市规划建设管理得到加强，城市面貌发生新变化。在深入开展“双创”和城市综合整治活动的基础上，加强城市规划管理，拉大城市框架，完善城市功能，提高城市规划和单体景观设计水平，突出城市亮点建设，使城市品牌和档次得到了进一步提升。建设了苏北路、中原东路，改造了人民路和昆吾路中段，拓宽了中原路西段、濮上路北段和胜利路西段，开工建设了西环路和北环路，中心广场和垂柳街等10条背街小巷进行了全面改造。城市供水供热管网改造和马颊河治理等完成了计划任务。城市绿化美化和居民居住条件进一步改善，绿化覆盖率达到42.6%，人均公共绿地面积达到15.8平方米，人均住房建筑面积达到24.5平方米。县城和小城镇建设步伐加快，全市城镇化率达到27.7%，提高了2.1个百分点。我市被国务院命名为国家历史文化名城。旅游业快速发展，全年接待游客206.6万人次，旅游总收入2.5亿元，分别比上年增长8.2%和49.7%。旅游景区开发取得新进展，戚城景区晋升为国家4A级景区。城市的环境优势正在转化为经济优势。

———社会事业全面发展，人民生活水平进一步提高。大力实施科教兴濮战略，加快教育发展和科技进步，加强人才队伍建设，增强了经济发展的人才和智力支持。组织实施各类科技项目110项，清丰县被命名为全国科技示范县，新增民营科技企业42家。教育事业不断进步，“普九”成果进一步巩固，职业教育快速发展，教育基础设施进一步完善，改造完成农村中小学D级危房23.7万平方米。高度重视人口和计划生育工作，大力开展争先创优活动，全市人口自然增长率达到4.98‰，完成了省下达的人口控制目标。以创建文化建设先进县为载体，全面加强农村文化建设，丰富了群众的文化生活。广泛开展全民健身运动，举办了濮阳市第六届运动会。疾病预防控制和医疗救治体系建设得到加强，市疾病控制中心检验楼、市紧急救援中心和市传染病医院门诊楼主体工程完工，市、县两级防疫站建设进展顺利，台前县新型农村合作医疗试点工作成效显著，城乡医疗设施和卫生条件不断改善。按照省统一部署和要求，完成了全市既往有偿供血人员普查任务。深入开展爱国卫生运动，社区卫生服务进一步深化。落实就业再就业扶持政策，全市新增就业人员4.2万人，城镇登记失业率达到3%，实现再就业2.8万人，其中“4050”人员达到7579人。完善社会保障体系，国有企业下岗职工基本生活费和企业离退休人员养老金按时足额发放，城市“低保”做到了应保尽保。国有企业下岗职工基本生活保障向失业保险并轨基本完成。人民生活水平继续提高，全市城镇居民人均可支配收入达到7091元，比上年增长12.1%；农民人均纯收入达到2186元，增长12.2%。高度重视安全生产工作，强化安全生产责任制，加强安全生产检查及专项整治，濮州大市场占压油气管线等重大事故隐患得到彻底整改，安全生产工作取得了明显成效，被评为2004年度全省安全生产工作先进市。关闭、取缔污染企业49家，环境保护工作进一步得到加强。加大农民工工资和工程款清欠力度，全部完成当年清欠目标。濮阳年鉴荣获首届中国地方志年鉴奖特等奖。第一次全国经济普查工作全面启动。土地管理、广播电视、民族宗教、外事侨务、审计、人防、地震、气象、保险、邮电通信、残疾人事业、国防教育、民兵预备役等工作都取得了新成绩。深入开展机关效能建设，行政效率和服务水平进一步提高。党风廉政建设取得了新成绩。精神文明和民主法制建设取得了新成效。加强社会治安综合治理，深入开展严打等专项活动和命案攻坚，社会治安防控能力进一步增强，被命名为全省社会治安综合治理先进市。认真做好信访工作，积极开展下访活动，社会大局保持稳定。

回顾和总结去年的工作，主要有以下六点体会和做法：

（一）把宏观调控作为新的机遇，千方百计加快发展步伐。面对国家实行宏观调控政策的新形势，我们坚持以发展为主题，全面理解和落实国家宏观调控政策，立足市情，准确定位，把国家加强宏观调控作为加快我市经济发展的重大机遇，适时调整工作思路，强力推进符合国家产业政策的重点项目建设，拉动了经济的增长。同时，根据濮阳现有基础和比较优势，坚持用改革的办法解决发展中的问题，用改革的办法促进发展。采取超常规措施，跳跃式发展，把濮阳经济做大做强，使全市各行各业的工作在全省晋位升级。在工作中，实施重点突破，整体推进，紧紧抓住对濮阳发展具有重要影响的大事、实事，一件一件地研究决策，一个一个地抓好落实。坚持实施责任制推进，明确任务，强化督导，促进了全市经济快速发展。

（二）把结构调整作为经济工作的主线，增强自主发展能力。经济结构不合理，结构单一，自主发展能力不强，是濮阳的基本市情。由于中央企业经济总量在我市经济中占的比重较大，全市经济发展受中央企业经济发展的影响很大，这种经济结构性的“软肋”，给我市快速发展带来了很大的影响。对此，我们立足长远，把结构调整和转变增长方式作为经济工作的主线，强力推进，增强经济的自主发展能力和向新的增长方式转变。主要是大力实施了以工兴市和对外开放主战略，加快非公有制经济和县域经济发展，使地方经济总量不断膨胀，经济规模不断增大。结构调整的成效初步显现，经济发展抗风险的能力日趋增强。

（三）把经济工作重心下移，着眼长远夯实发展基础。我市二元结构矛盾突出，农村经济薄弱，沿黄地区自然条件差，农民人均收入居全省后列。对此，我们坚持把解决“三农”问题作为各项工作的重中之重，认真落实中央一号文件精神以及中央、省一系列政策措施，进一步加大农业投入，着力加强农业基础设施建设。去年全市各项支农支出达到1.98亿元，比上年增长39.9%。为了统筹城乡经济发展，增强县域经济活力，制定了一系列简政放权的政策措施，从计划、财税、工商、土地等方面进一步加大了支持力度，取得了良好的效果。在全市发展大局上，坚持务实的态度和作风，不做表面文章，不追求一时的轰动效应，立足当前，着眼长远，超前谋划大项目，为濮阳今后的发展夯实基础。去年着力推进了全市项目库建设，积极运作了中原大化煤化工、2×35万千瓦燃气机组等一批大项目，这些项目的建设将对濮阳今后的发展产生重要作用。

（四）加快改革开放步伐，增强发展的动力和活力。我市基础差、底子薄、困难大、矛盾多，解决这些问题的关键是加快发展。要加快发展，必须把着力点放在深化改革和扩大开放上。在改革方面，注意把握改革的整体性和配套措施，使改革进一步深化。对国有企业改革坚持突出重点，整体推进，一企一策，在产权制度改革、法人治理结构建立、加强内控管理上下功夫，促使企业真正成为市场的主体。同时，计划、金融、财税、投资、价格以及社会保障制度、城镇住房制度等各方面改革加大了力度，取得了明显效果。实施对外开放主战略，大力开展招商引资活动。去年，我市出台了《关于加快发展开放型经济的决定》和《濮阳市招商引资奖励暂行办法》等政策措施，强力推动招商引资。抓住国内外特别是沿海地区产业转移的战略性机遇，以项目为载体，以优化环境为基础，充分利用两个市场、两种资源，大力引进境外和市外资金、技术、人才和管理经验，承接产业转移，发展濮阳。

（五）把提高人民群众生活水平作为根本出发点，坚持执政为民。我们时刻牢记党的宗旨，视群众利益高于一切，用实际行动践行“三个代表”重要思想，采取切实有效的措施，努力提高广大人民群众的生活水平。落实了粮食直补资金4503万元、良种补贴和农机补贴613万元、退耕还林粮食补助资金980万元，降低农业税率减征农业税7322万元，减轻了农民负担，增加了农民收入。全市亩均减负34.9元，人均减负40.4元。同时，出台了《关于进一步加强劳务输出工作的意见》，扩大劳务输出，外出务工农民达到37.2万人。多渠道增加职工和城镇居民收入。认真做好救灾工作，积极帮助黄河滩区受灾群众搞好毁坏房屋、水毁工程的维修与重建，确保受灾群众“有饭吃、有房住、有衣穿、有病能及时治疗、学生正常上学”。坚持开发式扶贫的方针，积极开展对口帮扶、定点扶贫，帮助农民群众多形式多渠道开展生产自救，实现脱贫。从维护广大市民的根本利益出发，扎扎实实地办好了18件实事。

（六）把优化环境作为促进发展的基础工程，增强对外吸引力。我们高度重视优化经济发展环境工作，始终把环境作为第一竞争点，作为生产力，不断采取强有力的措施进行优化。一方面，制定了一系列优惠政策，为投资者提供全方位服务。另一方面，采取有力措施，规范行政行为，整顿和规范市场经济秩序，共铸“诚信濮阳”，初步形成了让投资者放心、创业者舒心、劳动者安心的投资发展环境，极大地增强了我市的吸引力和凝聚力。

各位代表，过去一年成绩的取得，是省委、省政府和市委正确领导的结果，是全市人民共同奋斗的结果。在此，我代表市人民政府向全市工人、农民、知识分子、干部、人民解放军和武警官兵及各界人士致以崇高的敬意，表示衷心的感谢！

在肯定成绩的同时，我们也清醒地认识到，我市经济社会发展中还存在一些突出矛盾和问题。主要表现为：经济总量不大，实力不强；经济结构单一，地方工业规模偏小；农业基础薄弱，农民增收缓慢，特别是沿黄地区的内涝严重影响农业发展和农民增收；城镇化进程滞后，城镇产业发展不足；金融机构存贷反差较大，社会保障和再就业任务繁重，地方财政尤其是县乡财政困难等。对此，我们一定高度重视，并在今后的工作中认真加以解决。

二、2005年工作总体要求、奋斗目标和主要任务

今年政府工作的总体要求是：以邓小平理论和“三个代表”重要思想为指导，认真贯彻党的十六大、十六届三中、四中全会精神，坚持以科学发展观统领经济社会发展全局，以经济建设为中心，围绕全面建设小康社会的目标，大力实施以工兴市战略、科教兴濮战略、对外开放战略、可持续发展战略和品牌城市战略，加快经济结构调整，转变经济增长方式，加快工业化、城镇化，推进农业现代化，改善人民群众生活，发展各项社会事业，努力构建和谐社会，促进经济社会全面协调可持续发展。

今年我市经济社会发展的主要预期目标是：生产总值增长11%，全社会固定资产投资增长18%，地方财政收入增长12%，社会消费品零售总额增长12%，外贸出口增长15%，实际利用外资增长25%，居民消费价格总水平上涨5%，城镇居民人均可支配收入增长6%，农民人均纯收入增长5%，城镇新增就业人数3.5万人，城镇登记失业率控制在4.5%以内，人口自然增长率控制在6‰以内，工业企业经济效益综合指数、实现利润等质量和效益指标均要进一步提高。

为完成上述目标，今年要重点抓好十个方面的工作：

（一）大力实施以工兴市战略，进一步壮大经济实力。当前我市工业发展形势很好，但也面临着一些值得重视的问题。一方面是地方企业规模小、数量少、综合竞争能力不强；另一方面作为油气原料基地，没有形成产业链，缺少接续产品，资源优势没有充分发挥，影响了工业对经济的贡献率。因此，加快濮阳工业发展，必须走新型工业化道路，着力发展循环经济，推动工业增长方式的转变。一要壮大规模。就是继续坚持“抓大带小”两条腿走路。要千方百计为中原油田、中原乙烯、中原大化三大企业营造良好的外部环境，提供全方位的服务，促其加快技术改造与创新，搞好优势资源的精深加工，拉长产业和产品链条，带动地方经济发展。同时充分利用石油、天然气、乙烯、农副产品资源优势和劳动力优势，大力培育扶持地方中小企业发展壮大，增强地方经济发展后劲。二要调整结构。就是加快运用高新技术和先进适用技术改造提升传统产业，发展精深加工产品、终端产品和高档产品，提升产品档次。要集中力量抓好116个投资超千万元的重点工业项目建设，特别是要围绕油气资源优势，做大做强石化产业。目前，我们要利用本地的丰富石化原料，发挥资源优势，进行本地化资源开发和深加工，发展产业化，变弱势为强势，促进经济快速发展。要组织市发改委、国资委、企业服务局等部门抓紧研究规划一批油气资源及石化产品、玻璃产品、农副产品的深加工项目，对外招商，拉长产业链条，培育支柱产业。有关企业在向外地提供原料的同时，要多做工作，吸引原料加工企业到濮阳办厂，在我市形成产业链。三要转变增长方式。要着眼长远，整合资源，增加科技含量，形成规模效益，改变目前小企业分散建设、粗放经营模式，提高竞争力，规避市场风险。各县（区）都要加强工业园区的配套完善，搞好基础设施建设，使新项目向工业园区聚集。今后全市的重点工业项目，都要进入园区建设。四要加强调度运行。要进一步加强工业经济运行的监测、分析和指导，密切关注国内外市场变化，及时研究解决工业经济运行中出现的矛盾和问题，特别要针对煤电油运等生产要素十分紧张的严峻形势，强化组织调度，确保工业经济健康运行。

（二）进一步加强“三农”工作，促进农民继续增收。农民增收问题，关系农业和农村工作全局，关系农村社会稳定大局，始终是我们工作的重中之重。要认真贯彻中央、省委农村工作会议精神，全面落实中央和省各项支农政策，用工业化的理念发展农业，努力提高农业综合生产能力，实现农民增收。一要将稳定粮食生产与调整结构结合起来，把发展粮食生产的着力点放在应用科学技术、推广优良品种、提高单产上，确保全市粮食产量目标的完成。同时，要调整农业结构，扩大高效农作物种植，为农民增收提供足够的空间。二要落实增收减负等优惠政策。认真落实免征农业税、对种粮农民直接补贴、扩大良种补贴和农机具购置补贴等项政策，使农民得到更多的实惠。三要大力推进农业产业化经营和基地建设。重点扶持50户市级重点龙头企业建设，提高粮食及畜产品深加工转化能力，增强辐射带动能力。加强市农业园区建设，继续抓好160万亩优质专用小麦、40万亩优质水稻、90万亩优质玉米、30万亩出口型优质花生、5000亩鲜切花、30万亩尖椒及无公害蔬菜基地的开发工作。搞好优质畜产品生产加工基地建设，发展养殖小区，提高畜牧业的规模效益。四要切实加强农技推广体系建设，重点抓好清丰县试点工作。要建立健全农产品的标准、质量认证和检验监测体系，提高农产品质量和竞争力。五要加强农村基础设施和林业生态建设。加大农业综合开发和大型灌区配套建设力度，加快金堤河二期治理、地下水保护、渠村灌区配套和节水改造等项目的立项与建设进度，抓好黄河滩区治理工作，搞好农田水利建设、中低产田改造和农村道路改造等，改善农民生产生活条件。要围绕林纸一体化建设项目，突出抓好清丰、南乐30万亩光兆速生丰产林基地建设，抓好退耕还林、重点地区防护林、防沙治沙等工程，加快林业产业化步伐。六要狠抓劳务输出。认真搞好劳动力转移培训和管理工作，年内完成40万人的劳务输出任务，增加农民工资性收入。七要进一步加大扶贫工作力度，强力实施产业化扶贫、科技扶贫和开发式扶贫，使更多的群众摆脱贫困，走上富裕之路。

（三）强力推进重点项目建设，千方百计扩大固定资产投资。要抓住国家加大对薄弱环节和结构调整支持力度的机遇，优化投资结构，加大工作力度，开工建设一批影响濮阳经济发展全局的重大项目。今年要着力抓好产业和基础设施方面的150个重点项目，概算总投资190亿元，年内计划完成投资100亿元。开工建设濮范高速公路、中原大化30万吨甲醇、中原乙烯28万吨改造、贝利石化120万吨炼油装置扩建等大项目。继续抓好濮信高速公路濮阳段、林纸一体化项目、2×20万千瓦热电机组、中原大化3万吨三聚氰胺、范县陆集村台建设、地下水源给水工程、温州商贸城等重点续建项目。做好市甲醇厂40万吨甲醇项目、2×35万千瓦燃气机组项目的前期工作。要根据国家、省的产业政策和投资方向，进一步搞好项目库建设，调整充实一批市场潜力大、科技含量高、带动能力强的项目，尤其要运作好今后2至3年内可能开工建设的大项目。同时，今年市政府要办好与全市人民群众生产生活息息相关的12件实事：（1）完成城市用水黄河取水口改建工程，让广大市民吃上安全水、放心水；（2）开工建设金堤河下游支沟口闸站项目，解决范县、台前县内涝问题；（3）基本完成农村中小学D级危房改造，解决农村中小学安全隐患问题；（4）建设农村安全饮水工程，解决苦水区36个村4万农民安全饮水问题；（5）完成市社会福利院建设，使残疾弃婴等弱势群体的生活得到保障；（6）完成昆吾花园南区小学建设，缓解城区小学入学压力；（7）完成市人才交流中心建设，为人才开发和就业提供平台；（8）完成10条背街小巷改造，方便市民出行；（9）建设6座垃圾中转站、7座公厕，完善城市环境卫生功能；（10）完成胜利路、开州路雨水泵站建设和行政南小区污水泵站改造，开工建设濮水河截污工程，完善城市基础设施功能；（11）完成市县传染病防治病区建设，维护人民群众身体健康；（12）支持城市社区卫生服务中心（站）建设，为市民提供经济、方便的综合医疗卫生服务。

为完成今年的项目建设，要实行重点项目重点抓，一个项目一个项目地落实责任，落实资金。要下大力气改善建设环境，及时协调解决项目审批、征地拆迁、安置补偿、配套资金等问题，确保工程进度和质量。要拓宽融资渠道，引导更多的资金进入经济建设领域。要加大建设项目的稽察和监管力度，提高投资效益。

（四）加大招商引资力度，发展壮大非公有制经济。一要全面落实《中共河南省委河南省人民政府关于加快发展开放型经济的若干意见》，兑现政策，严格奖惩，加大招商引资力度，扩大产品出口。二要有大手笔，大动作。按照“盯住、靠上、抓牢、办成”的原则，在巩固招商引资成果的基础上，要扩大视野，瞄准世界500强，采取超常措施，克服一切困难，力争招大商、引大资。同时，要强化“市外就是外”的观念，紧紧盯住与开发濮阳资源相关的国内企业强手和沿海民营大企业，引进规模大、技术含量高、带动能力强的大型企业集团。三要创造性地开展招商引资工作。要创新招商方式，“走出去”，“请进来”，努力扩大招商引资成果。全力办好世界张氏总会第二届恳亲大会，并以此大力开展乡情招商、亲情招商、团队招商，力争有更多的项目落户濮阳。要积极参加国家、省举办的大型投资贸易洽谈活动，同时精心组织赴浙江的招商活动。四要加强已签约重大项目的落实。重点抓好去年“长三角”和“珠三角”招商引资活动中52个签约项目的跟踪问效，千方百计争取资金到位、项目开工建设。五要进一步落实外贸出口政策，巩固现有出口市场。要抓住今年取消纺织品配额的机遇，扩大纺织品出口。同时要转变外贸出口方式，多上一些高科技产品和终端产品，发展特色出口商品，形成新的创汇优势。要继续做好外经工作，保持在全省领先位置。要加快高新区的建设和发展，以基础设施建设和招商引资为重点，把引进大项目和高新技术项目作为主攻方向，努力把高新区建成我市对外开放的窗口和展示城市形象的窗口，建成新型产业发展的基地和濮阳发展后劲的动力基地，在全市新一轮快速发展中发挥示范带动作用。

非公有制经济是我市经济发展最具潜力的经济增长点，必须摆到更加突出的位置来抓。要在巩固现有非公有制经济的基础上，通过加大招商引资力度，激活民间资金，加快非公有制经济的发展。深入贯彻落实市委、市政府《关于进一步促进非公有制经济发展的意见》，切实把中央和省扶持非公有制经济发展的各项政策措施落到实处，为非公有制企业创造平等竞争、一视同仁的法制环境、政策环境和市场环境。要进一步放宽非公有制资本进入基础设施、垄断行业、公用事业以及法律法规没有禁止的其他行业和领域；鼓励非公有资本参与国有和集体企业改制和重组；切实保护私有财产权和非公有制企业的合法权益；推动非公有制企业体制、机制、技术和管理创新，不断提高自身素质，为非公有制企业做大做强搞好全方位服务。

（五）深化改革，增强经济发展的动力和活力。一要继续以产权制度改革为重点，积极稳妥地推进国有企业改革。坚持区别对待、分类指导的原则，支持主业突出、核心竞争力较强的企业做大做强，使劣势企业退出市场。今年要重点抓好市直21户国有企业的改革改制工作。企业改革不能只走破产这一条路子，要因企制宜，一厂一策，采取收购、兼并、控股、参股、租赁、承包等多种形式，大胆地尝试和探索，千方百计放开搞活国有中小企业。要严格改制程序，规范操作，避免国有资产流失。积极做好不良债权回购和处置工作，切实减轻国有企业包袱。要健全国有资产监管体系，完善国有资产出资人制度、授权监管范围和授权经营制度、国有企业负责人经营业绩考核制度及薪酬分配制度，向市属重点国有企业委派监事会，加强国有资产监管。做好清产核资工作，探索国有资产保值增值的模式。市财政要列出专项改制资金，大力扶持国有企业改革。二要继续深化粮食流通体制改革。要在去年完成国有粮食企业职工分流任务的基础上，以产权制度改革为核心，以建立现代企业制度为方向，按照“政企分开、股份改制、优化重组、自负盈亏”的原则，对国有粮食购销企业进行资产重组，完善法人治理结构，实行集团化发展、规模化经营。三要进一步深化农村信用社体制改革。重点抓好农村信用社增资扩股、清收不良贷款等工作，使资本充足率、不良贷款率达到中央银行规定的要求，确保今年上半年专项央行票据如期到位。认真落实国家有关税费减免政策，取消针对农村信用社的各种歧视性规定。完善基层农村信用社法人治理结构，转换经营机制，健全内控机制，增强服务功能。四要做好其他领域的改革。进一步深化投资体制改革，规范政府投资范围和行为，落实企业投资自主权。按照省政府的要求，加快公用事业市场化改革。有计划、有步骤地推进供水、供热、公交、污水处理、垃圾处理等行业市场化，通过活化产权，培育市场竞争的主体，真正建立起投资多元化、运营市场化、管理科学化、发展产业化、服务社会化的公用事业管理新体制。积极推进事业单位改革，在全部实行聘用制的基础上，规范人事管理，严格推行竞争上岗和双向选择制度。搞好交通、城建、水利等建管体制改革和科教文卫等社会事业领域的改革。推进统计改革，完善核算体系和方法，建立健全经济运行监测体系。

（六）积极推进城镇化进程，加快城市经济与县域经济发展。要进一步加强城市建设和管理工作，重点是加强规划的统一管理，维护规划的严肃性，提高规划执行的刚性，确保城市规范有序发展，努力打造品牌城市。首先，要加大城市规划管理力度。城市规划由市级规划行政主管部门统一管理，做到“规划一张图，审批一支笔”。我市新的城市总体规划纲要已通过专家评审，要按照规划纲要，抓紧编制城市专业规划、详细规划和控制性规划。其次，要进一步提高城市品位和档次。要在规划指导下，解决城市建设水平低的问题，完善基础设施建设，提高城市建设品位，增加标志性建筑物，形成景观，突出濮阳特色。第三，要强化城市管理。加大城市综合整治力度，治理脏乱差，清理乱搭乱建，解决垃圾围城和日产日清问题，搞好绿化、净化、亮化、美化，加强市容市貌建设。与此同时，5个县城建设要根据规划全面推进，完善功能，加快发展。小城镇建设要在规划指导下，因地制宜，突出特色，注重实效。

要大力发展城市经济。进一步强化城市产业支撑，重点培植支柱产业，以产业和品牌支撑城市化进程。上半年，要研究制定出我市城市经济发展规划，尽快付诸实施。通过招商引资大上项目，培育新的增长点。要组织市发改委、国资委、商务局、农业局、企业服务局等部门按照职能，在抓好全市发展工作的同时，担负起市本级经济发展主力军的重要责任，成为市本级招商引资和增加新的经济增长点的主体，采取有效措施，大力引进项目，狠抓落实，加快城市经济发展。要在市高新区划出一定区域，用于市级工业新项目的建设。为了加快城镇化进程，要积极推进城镇户籍制度改革，降低门槛，膨胀城市人口规模。要大力发展劳动密集型产业，创造就业岗位，增强城市吸纳能力。要用先进的理念和现代技术改造提升传统服务业，大力发展信息、金融、中介等现代服务业和社区服务、教育培训、文化体育等需求潜力大的产业。要大力发展现代物流业，积极培育现代大型物流集团。要积极发展旅游业，创新宣传促销方式，培育旅游品牌，将旅游业培育成新的经济产业。

要进一步加快县域经济发展。认真落实国家和省支持县域经济发展的政策，简政放权，放手发展。今年，国家和省将重点支持县城污水处理、垃圾处理等基础设施建设，各级各有关部门要“跑部进厅”，争取给予支持。要坚持项目支撑，发挥资源优势，加大招商引资力度，不断引进资金、技术、人才和先进管理经验，培育一批技术含量较高的产品，培育和壮大各具特色的支柱产业，打造一批竞争力强的县域经济骨干企业，使建材、石化、玻璃、塑编等产业，形成具有比较优势的产业集群。抓好利用国债资金项目，规划建设一批县域综合农业生产资料市场、乡镇生产资料配送中心和网点。完善县域经济发展激励机制，年底要对两年来县域经济发展情况进行全面总结，对发展较快的县（区）予以表彰和奖励。

（七）加强财税金融工作，更好地支持经济社会发展。要牢固树立发展经济、培植税源的思想，采取有力措施，加强财源建设和税收征管，促进财政增收。要依法实行综合治税，应收尽收，堵塞“跑、冒、滴、漏”，确保财政收入稳定增长。要加强土地、广告、污水处理费、市政设施经营权转让等非税收入的收缴管理。同时，积极争取中央、省财政转移支付，增加可用财力。完善县乡财政体制，全面推行“乡财县管”，落实“三奖一补”政策，缓解县乡财政困难。要按照“分级管理、分灶吃饭”的原则，合理安排财政支出，保证农业、教育、科技、社会保障等法定和重点支出需要。要继续推进部门预算、国库集中收付、政府采购和“收支两条线”等规定，严格支出管理，强化财政财务监督，加大审计执法力度。加大政府投资评审力度，严禁大手大脚、铺张浪费，提高财政资金的使用效益。金融机构要用足用活信贷政策，加强内部管理，增加信贷投入，加强银企合作，确保重点企业、重点建设项目的资金需求，并扩大对中小企业、民营企业和县域经济发展的贷款规模。各级政府要加大对金融部门的支持，搞好服务，为金融机构创造良好的经营环境。

（八）坚持科教兴濮和可持续发展战略，统筹发展各项社会事业。要大力实施科教兴濮战略，加大科技创新力度，建立和完善企业技术创新机制，搞好科技成果的引进、示范和推广，提高科技成果转化率。要进一步完善农村科技信息“村村通”和“户户通”工程，搞好基层科技服务体系建设。坚持教育优先发展的战略地位，抓好基础教育，扶持农村教育，发展高中阶段教育、职业教育、成人教育和特殊教育，办好高等教育，加快教育信息化建设步伐。大力实施人才强市战略，加强公务员队伍和专业技术人才队伍建设，做好人才资源开发和利用工作，全面提升人才队伍素质。牢固树立人均观念，坚持一把手负总责、亲自抓，切实加强人口与计划生育工作，大力开展争先创优活动，稳定低生育水平，提高出生人口素质。广泛开展爱国卫生运动，完善公共卫生体系，加快农村卫生事业发展，提高城乡居民健康水平。要促进文化事业和文化产业发展，抓好市文化中心、市电视编播楼建设，开展积极向上的群众性文化活动。加强对历史文化名城和历史遗产的保护和利用。完善体育基础设施，广泛开展群众性体育活动和全民健身运动。继续加大环境保护力度，确保城市饮用水源水质稳定达标、城市空气质量进一步提高。抓好市辖流域水污染防治，河流出境断面水质达到责任目标要求。按照省政府要求，切实做好城镇污水处理费和城市垃圾处理费的征缴工作。完善资源有偿使用机制，合理保护和利用自然资源，建设节约型社会，促进人与自然和谐发展。深化教育、医疗、住房、药品、水、天然气等方面的价格改革，保持物价平稳运行，维护社会稳定。要认真做好第一次全国经济普查工作，为国家和濮阳经济发展摸清家底，打好基础。继续做好广播电视、外事侨务、民族宗教、人防、保险、邮电通信、气象、史志、地震、残疾人事业等工作。

（九）坚持以人为本，努力构建社会主义和谐社会。一要继续实行积极的就业政策。要落实就业再就业的各项优惠政策，把财政投入、小额贷款、免费培训和税费减免等支持再就业政策落到实处。要大力发展服务业和劳动密集型企业，努力创造更多的就业岗位。建立健全就业服务和再就业援助体系，重点帮助“4050”人员和城市低保对象等困难群体就业。要统筹各方面就业工作，继续做好大学生、复员转业军人、城镇新增劳动力和农村富余劳动力的就业和安置工作。二要切实做好社会保障工作。继续巩固“两个确保”，规范和完善城市低保工作，做到应保尽保。按照省里要求，今年6月底要完成企业职工基本养老保险市级统筹的目标。要加大对各类参保企业社会保险费的征缴力度，扩大征缴面，提高征缴率。对企业退休人员管理服务进社区要进行探索，总结经验，认真做好。鼓励有条件的地方建立农村最低生活保障制度，更加关注、关心弱势群体，使城乡困难群众的基本生活得到保障。三要解决关系人民群众切身利益的问题。继续整顿和规范市场经济秩序，严厉打击制售假冒伪劣食品、药品等违法活动，保护群众生命安全和身体健康。妥善安排受灾群众的生产生活。认真解决建设领域拖欠工程款和农民工工资问题，确保完成年度清欠目标任务，决不允许发生新的拖欠，保障农民工的合法权益。四要努力打造“平安濮阳”。全面落实社会治安综合治理各项措施，深入开展严打和命案攻坚，强化社会治安管理，推进社会治安防控体系建设。高度重视安全生产工作，落实安全生产责任制，强化基础设施建设，构筑安全生产长效机制，警钟长鸣，常抓不懈，坚决遏制重特大事故的发生。要正确处理新形势下人民内部矛盾，健全预防、化解各种矛盾和纠纷的机制，努力做好人民来信来访工作，维护正常社会秩序和社会稳定。

（十）进一步加强精神文明建设，推进民主法制进程。加强精神文明建设。全面落实《公民道德建设实施纲要》，深入开展社会公德、职业道德和家庭美德教育，在全社会形成良好道德风尚。进一步发扬光大焦裕禄精神、红旗渠精神。继续加强未成年人思想道德建设和大学生思想政治建设。广泛开展“弘扬民族精神、实现中原崛起”主题教育活动。深入开展群众性精神文明创建活动。要搞好“双创”工作，严格按照《全国文明城市测评体系》标准，分解目标，落实责任，确保“全国文明城市创建工作先进城市”称号，力争“全国文明城市”创建有新的业绩。加强全社会诚信体系建设，倡导诚实守信的良好社会风气。积极开展科技、文化、体育、法律、卫生进社区和科技、教育、卫生“三下乡”活动，形成健康向上、文明和谐的社会环境。

加强民主法制建设。坚持依法治市，贯彻“四五”普法和“二五”依法治市工作规划，加强以宪法为核心的法制宣传，提高全体公民尤其是公职人员的法律素质。严格执行市人大及其常委会通过的各项决定和决议，自觉接受其工作监督和法律监督，支持人大代表依法行使职权。加强与人民政协的协商，接受人民政协的民主监督。认真办理人大代表建议和政协提案。广泛听取各民主党派和工商联的意见，充分发挥工会、共青团、妇联等人民团体的桥梁纽带作用。积极开展法律援助，依法保护妇女儿童、残疾人和未成年人的合法权益。巩固和扩大基层民主，完善村民自治、社区民主管理和企业民主管理制度，做好第五届村民委员会换届工作。

积极支持部队和国防建设。深入开展国防教育，健全国防动员体系。继续开展争创“双拥模范城”活动。关心支持驻濮解放军、武警、消防部队和民兵预备役建设，做好军队转业干部、退役士兵安置和拥军优属工作，增强军政军民团结。

三、努力提高政府工作水平

2005年，是全面完成“十五”目标、为“十一五”发展打好基础的关键一年。各级政府必须适应新的形势，把贯彻落实科学发展观作为政府工作的主线，进一步提高行政能力和工作水平。

要进一步转变政府职能。在继续履行好政府经济调节、市场监管职能的同时，更加注重强化社会管理和公共服务职能，真正把政府职能转到为市场主体服务和创造良好发展环境上来，把有限的财力物力等资源向公共服务方面倾斜，把更多的时间精力放到推进社会发展和解决民生问题上。增强服务意识，改进服务方式，提高服务效率，积极推进电子政务。深化县、乡两级政务公开，全面推行市级政务公开，增强政府工作透明度，提高政府公信力。

要努力提高经济社会管理水平。进一步改进经济管理的方式方法，自觉按市场经济规律办事，努力提高驾驭社会主义市场经济的能力，处理好速度与质量、结构、效益之间的关系，坚持实事求是，不盲目攀比速度，在产业结构优化、土地集约利用、减少污染物排放、节能降耗、降低生产成本等方面狠下功夫，切实把工作着力点放在提高经济增长的质量和效益上。立足当前、谋划长远，既要着力解决好当前经济运行中的问题，防止经济发展出现大的波动，又要谋划好中长期发展，做好“十一五”计划和2020年远景规划编制工作。充分发扬民主，善于集中民智，推进决策民主化和科学化，健全重大问题集体决策制度、专家咨询制度、社会公示和听证制度，提高公众参与度。进一步改进社会事业管理，依法管理和规范社会组织、社会事务，协调处理好各种利益关系，促进社会公平和正义。建立健全社会预警体系和突发事件应急机制，提高保障公共安全和应对突发事件的能力。

要全面推进依法行政。强化依法行政观念，严格按照法定权限和程序行使权力、履行职责。深入贯彻行政许可法和依法行政实施纲要，继续深化行政审批制度改革。进一步规范行政执法主体，加强和改进行政执法，做到严格执法、公正执法、文明执法。强化执法监督，严格实行行政执法责任制和执法过错责任追究制，认真解决有法不依、执法不公和多头执法、执法扰民等群众反映强烈的问题，严厉打击吃拿卡要等行为。进一步加强行政监察工作，继续深入开展机关效能建设，加大对“三强四霸”等危害经济发展环境行为的查处力度，优化经济发展环境。严格落实“五不准”规定，提高行政效率和服务水平。

要大力加强政风建设。牢固树立正确的政绩观，始终做到人民利益高于一切。多做打基础、管长远、惠及百姓的工作，坚决杜绝劳民伤财的“政绩工程”、“形象工程”。加快建立和完善新的经济社会发展综合评价体系和干部政绩考核评价体系。大兴求真务实之风，继续下大力气精简会议、文件，改进会风、文风，最大限度减少应酬和不必要的事务性活动。拿出更多的时间开展调查研究，深入了解社情民意，多为群众办实事、做好事。强化责任意识，狠抓工作落实，敢于坚持原则，敢于触及矛盾，敢于批评错误，反对好人主义，反对敷衍塞责，反对推诿扯皮。牢记“两个务必”，始终保持谦虚谨慎、不骄不躁、艰苦奋斗的优良作风。坚持廉洁从政、从严治政，旗帜鲜明地惩治腐败。

各位代表，我们面临的任务艰巨而繁重。让我们高举邓小平理论和“三个代表”重要思想伟大旗帜，在省委、省政府和市委的领导下，紧紧依靠全市人民，发奋图强，扎实工作，为全面实现濮阳经济振兴、社会和谐、人民富足而努力奋斗！
